# Community poverty level influences time to first pediatric rheumatology appointment in Polyarticular Juvenile Idiopathic Arthritis

**DOI:** 10.1186/s12969-021-00610-5

**Published:** 2021-08-14

**Authors:** Nayimisha Balmuri, William Daniel Soulsby, Victoria Cooley, Linda Gerber, Erica Lawson, Susan Goodman, Karen Onel, Bella Mehta, for the CARRA Registry Investigators,, N. Abel, K. Abulaban, A. Adams, M. Adams, R. Agbayani, J. Aiello, S. Akoghlanian, C. Alejandro, E. Allenspach, R. Alperin, M. Alpizar, G. Amarilyo, W. Ambler, E. Anderson, S. Ardoin, S. Armendariz, E. Baker, I. Balboni, S. Balevic, L. Ballenger, S. Ballinger, N. Balmuri, F. Barbar-Smiley, L. Barillas-Arias, M. Basiaga, K. Baszis, M. Becker, H. Bell-Brunson, E. Beltz, H. Benham, S. Benseler, W. Bernal, T. Beukelman, T. Bigley, B. Binstadt, C. Black, M. Blakley, J. Bohnsack, J. Boland, A. Boneparth, S. Bowman, C. Bracaglia, E. Brooks, M. Brothers, A. Brown, H. Brunner, M. Buckley, M. Buckley, H. Bukulmez, D. Bullock, B. Cameron, S. Canna, L. Cannon, P. Carper, V. Cartwright, E. Cassidy, L. Cerracchio, E. Chalom, J. Chang, A. Chang-Hoftman, V. Chauhan, P. Chira, T. Chinn, K. Chundru, H. Clairman, D. Co, A. Confair, H. Conlon, R. Connor, A. Cooper, J. Cooper, S. Cooper, C. Correll, R. Corvalan, D. Costanzo, R. Cron, L. Curiel-Duran, T. Curington, M. Curry, A. Dalrymple, A. Davis, C. Davis, C. Davis, T. Davis, F. De Benedetti, D. De Ranieri, J. Dean, F. Dedeoglu, M. DeGuzman, N. Delnay, V. Dempsey, E. DeSantis, T. Dickson, J. Dingle, B. Donaldson, E. Dorsey, S. Dover, J. Dowling, J. Drew, K. Driest, Q. Du, K. Duarte, D. Durkee, E. Duverger, J. Dvergsten, A. Eberhard, M. Eckert, K. Ede, B. Edelheit, C. Edens, C. Edens, Y. Edgerly, M. Elder, B. Ervin, S. Fadrhonc, C. Failing, D. Fair, M. Falcon, L. Favier, S. Federici, B. Feldman, J. Fennell, I. Ferguson, P. Ferguson, B. Ferreira, R. Ferrucho, K. Fields, T. Finkel, M. Fitzgerald, C. Fleming, O. Flynn, L. Fogel, E. Fox, M. Fox, L. Franco, M. Freeman, K. Fritz, S. Froese, R. Fuhlbrigge, J. Fuller, N. George, K. Gerhold, D. Gerstbacher, M. Gilbert, M. Gillispie-Taylor, E. Giverc, C. Godiwala, I. Goh, H. Goheer, D. Goldsmith, E. Gotschlich, A. Gotte, B. Gottlieb, C. Gracia, T. Graham, S. Grevich, T. Griffin, J. Griswold, A. Grom, M. Guevara, P. Guittar, M. Guzman, M. Hager, T. Hahn, O. Halyabar, E. Hammelev, M. Hance, A. Hanson, L. Harel, S. Haro, J. Harris, O. Harry, E. Hartigan, J. Hausmann, A. Hay, K. Hayward, J. Heiart, K. Hekl, L. Henderson, M. Henrickson, A. Hersh, K. Hickey, P. Hill, S. Hillyer, L. Hiraki, M. Hiskey, P. Hobday, C. Hoffart, M. Holland, M. Hollander, S. Hong, M. Horwitz, J. Hsu, A. Huber, J. Huggins, J. Hui-Yuen, C. Hung, J. Huntington, A. Huttenlocher, M. Ibarra, L. Imundo, C. Inman, A. Insalaco, A. Jackson, S. Jackson, K. James, G. Janow, J. Jaquith, S. Jared, N. Johnson, J. Jones, J. Jones, J. Jones, K. Jones, S. Jones, S. Joshi, L. Jung, C. Justice, A. Justiniano, N. Karan, K. Kaufman, A. Kemp, E. Kessler, U. Khalsa, B. Kienzle, S. Kim, Y. Kimura, D. Kingsbury, M. Kitcharoensakkul, T. Klausmeier, K. Klein, M. Klein-Gitelman, B. Kompelien, A. Kosikowski, L. Kovalick, J. Kracker, S. Kramer, C. Kremer, J. Lai, J. Lam, B. Lang, S. Lapidus, B. Lapin, A. Lasky, D. Latham, E. Lawson, R. Laxer, P. Lee, P. Lee, T. Lee, L. Lentini, M. Lerman, D. Levy, S. Li, S. Lieberman, L. Lim, C. Lin, N. Ling, M. Lingis, M. Lo, D. Lovell, D. Lowman, N. Luca, S. Lvovich, C. Madison, J. Madison, S. Magni Manzoni, B. Malla, J. Maller, M. Malloy, M. Mannion, C. Manos, L. Marques, A. Martyniuk, T. Mason, S. Mathus, L. McAllister, K. McCarthy, K. McConnell, E. McCormick, D. McCurdy, P. Mc Curdy Stokes, S. McGuire, I. McHale, A. McMonagle, C. McMullen-Jackson, E. Meidan, E. Mellins, E. Mendoza, R. Mercado, A. Merritt, L. Michalowski, P. Miettunen, M. Miller, D. Milojevic, E. Mirizio, E. Misajon, M. Mitchell, R. Modica, S. Mohan, K. Moore, L. Moorthy, S. Morgan, E. Morgan Dewitt, C. Moss, T. Moussa, V. Mruk, A. Murphy, E. Muscal, R. Nadler, B. Nahal, K. Nanda, N. Nasah, L. Nassi, S. Nativ, M. Natter, J. Neely, B. Nelson, L. Newhall, L. Ng, J. Nicholas, R. Nicolai, P. Nigrovic, J. Nocton, B. Nolan, E. Oberle, B. Obispo, B. O’Brien, T. O’Brien, O. Okeke, M. Oliver, J. Olson, K. O’Neil, K. Onel, A. Orandi, M. Orlando, S. Osei-Onomah, R. Oz, E. Pagano, A. Paller, N. Pan, S. Panupattanapong, M. Pardeo, J. Paredes, A. Parsons, J. Patel, K. Pentakota, P. Pepmueller, T. Pfeiffer, K. Phillippi, D. Pires Marafon, K. Phillippi, L. Ponder, R. Pooni, S. Prahalad, S. Pratt, S. Protopapas, B. Puplava, J. Quach, M. Quinlan-Waters, C. Rabinovich, S. Radhakrishna, J. Rafko, J. Raisian, A. Rakestraw, C. Ramirez, E. Ramsay, S. Ramsey, R. Randell, A. Reed, A. Reed, A. Reed, H. Reid, K. Remmel, A. Repp, A. Reyes, A. Richmond, M. Riebschleger, S. Ringold, M. Riordan, M. Riskalla, M. Ritter, R. Rivas-Chacon, A. Robinson, E. Rodela, M. Rodriquez, K. Rojas, T. Ronis, M. Rosenkranz, B. Rosolowski, H. Rothermel, D. Rothman, E. Roth-Wojcicki, K. Rouster-Steven, T. Rubinstein, N. Ruth, N. Saad, S. Sabbagh, E. Sacco, R. Sadun, C. Sandborg, A. Sanni, L. Santiago, A. Sarkissian, S. Savani, L. Scalzi, L. Schanberg, S. Scharnhorst, K. Schikler, A. Schlefman, H. Schmeling, K. Schmidt, E. Schmitt, R. Schneider, K. Schollaert-Fitch, G. Schulert, T. Seay, C. Seper, J. Shalen, R. Sheets, A. Shelly, S. Shenoi, K. Shergill, J. Shirley, M. Shishov, C. Shivers, E. Silverman, N. Singer, V. Sivaraman, J. Sletten, A. Smith, C. Smith, J. Smith, J. Smith, E. Smitherman, J. Soep, M. Son, S. Spence, L. Spiegel, J. Spitznagle, R. Sran, H. Srinivasalu, H. Stapp, K. Steigerwald, Y. Sterba Rakovchik, S. Stern, A. Stevens, B. Stevens, R. Stevenson, K. Stewart, C. Stingl, J. Stokes, M. Stoll, E. Stringer, S. Sule, J. Sumner, R. Sundel, M. Sutter, R. Syed, G. Syverson, A. Szymanski, S. Taber, R. Tal, A. Tambralli, A. Taneja, T. Tanner, S. Tapani, G. Tarshish, S. Tarvin, L. Tate, A. Taxter, J. Taylor, M. Terry, M. Tesher, A. Thatayatikom, B. Thomas, K. Tiffany, T. Ting, A. Tipp, D. Toib, K. Torok, C. Toruner, H. Tory, M. Toth, S. Tse, V. Tubwell, M. Twilt, S. Uriguen, T. Valcarcel, H. Van Mater, L. Vannoy, C. Varghese, N. Vasquez, K. Vazzana, R. Vehe, K. Veiga, J. Velez, J. Verbsky, G. Vilar, N. Volpe, E. von Scheven, S. Vora, J. Wagner, L. Wagner-Weiner, D. Wahezi, H. Waite, J. Walker, H. Walters, T. Wampler Muskardin, L. Waqar, M. Waterfield, M. Watson, A. Watts, P. Weiser, J. Weiss, P. Weiss, E. Wershba, A. White, C. Williams, A. Wise, J. Woo, L. Woolnough, T. Wright, E. Wu, A. Yalcindag, M. Yee, E. Yen, R. Yeung, K. Yomogida, Q. Yu, R. Zapata, A. Zartoshti, A. Zeft, R. Zeft, Y. Zhang, Y. Zhao, A. Zhu, C. Zic

**Affiliations:** 1 Hospital for Special Surgery, New York, NY USA; 2 Weill Cornell Medicine, New York, NY USA; 3 University of California, San Francisco, San Francisco, CA USA

## Abstract

**Background:**

The impact of social determinants of health on children with polyarticular juvenile idiopathic arthritis (pJIA) is poorly understood. Prompt initiation of treatment for pJIA is important to prevent disease morbidity; however, a potential barrier to early treatment of pJIAs is delayed presentation to a pediatric rheumatologist. We examined the impact of community poverty level, a key social determinant of health, on time from patient reported symptom onset to first pediatric rheumatology visit among pJIA patients enrolled in the Childhood Arthritis and Rheumatology Research Alliance (CARRA) Registry.

**Methods:**

This is a cohort study of pJIA patients in the CARRA registry who lived in the United States from July 2015–February 2020. The primary exposure was community poverty level derived by geocoding patient addresses. The primary outcome was time to first rheumatology appointment. Kaplan-Meier analysis was performed to analyze time to first rheumatologist visit, stratified by community poverty and family income. Log-rank tests were used to identify differences between groups. Adjusted cox proportional-hazards models were used to determine the relationship between community poverty level and time from onset of disease symptoms to date first seen by rheumatologist.

**Results:**

A total of 1684 patients with pJIA meeting study inclusion and exclusion criteria were identified. Median age of onset of pJIA was 7 years (IQR 3, 11), 79% were female, 17.6% identified as minority race and/or ethnicity, and 19% were from communities with ≥20% community poverty level. Kaplan-Meier analysis by community poverty level (< 20% vs ≥20%) yielded no significant differences with time to initial presentation to a pediatric rheumatologist (*p* = 0.6). The Cox proportional hazards model showed that patients with ≥20% community poverty level were 19% less likely (adjusted HR 0.81, 95% CI 0.67–0.99, *p* = 0.038) to be seen by a rheumatologist compared to patients with < 20% community poverty level, at the same time point, after adjusting for sex, race/ethnicity, insurance, education level, morning stiffness, RF status, and baseline CHAQ.

**Conclusion:**

In this study of pJIA patients in the CARRA registry, increased community poverty level is associated with longer time to presentation to a pediatric rheumatologist after symptom onset.

## Background

Juvenile idiopathic arthritis (JIA) includes a heterogeneous group of chronic childhood inflammatory arthritides that can lead to significant morbidity through damaging joints and impairing both physical function and growth. Rheumatoid factor (RF) positive and RF negative polyarticular JIA (pJIA) are two forms of arthritis that affect 5 or more joints. Among subtypes of JIA, children with pJIA are more likely to experience prolonged periods of active disease [1] with an increased risk for a relapsing and remitting course throughout the lifespan [2]. Irreparable damage to the joints and surrounding tissue is known to occur early in the course of disease in as many as 60% of patients with pJIA [3, 4]. In order to prevent this damage, it is imperative to bring disease under control as rapidly as possible underscoring the importance for a child with new-onset pJIA to present to a pediatric rheumatologist early on in the disease course [5].

Social determinants of health (SDH) such as access to public or private health insurance, guardian’s level of education, family income, and poverty level have all been associated with barriers to subspecialty follow up in children with chronic diseases in the United States [6, 7]. As SDH are not consistently reported in registries, area-based socioeconomic measures can be used as a proxy to provide insight into patients’ socioeconomic status. Features of community deprivation such as poverty are associated with sparse community resources needed for good health [8] with poor health outcomes consistently reported in communities where ≥20% of the population lives below the poverty level [9–11]. Delayed diagnosis and presentation to a rheumatologist has been described in adult rheumatoid arthritis [12–14] and reported in international cohorts of patients with juvenile idiopathic arthritis [15–17]. However, the effect of community poverty, a key social determinant of health, on time to presentation to a pediatric rheumatologist has not been described.

Evaluating disparities in delays to care is an important initial step toward identifying ways to overcome barriers to equitable care and to improve disease outcomes. In this study, we aimed to examine the effect of community poverty on time to first pediatric rheumatology visit among pJIA patients enrolled in the Childhood Arthritis and Rheumatology Research Alliance (CARRA) Registry, a large cohort of pJIA patients from North America.

## Methods

### Study design

We analyzed baseline data collected from the pJIA cohort in the CARRA Registry. The CARRA Registry includes data from children with rheumatic diseases from 70 sites in North America with all but four of these centers located in the United States (US). Patients diagnosed with pJIA prior to age 16 are enrolled into the Registry by a pediatric rheumatologist before the age of 21. Research personnel enter pertinent data from each CARRA site at the time of initial and follow up patient visits. Data utilized for this study were collected from April 2015 through February 2020.

### Analytical sample

Inclusion criteria were US residency and pJIA diagnosis with recorded ≥5 joints involved in first 6 months of disease. Exclusion criteria were invalid US zip code and additional diagnosis of systemic inflammatory or autoimmune disease. Approval for exemption was obtained from the Hospital for Special Surgery and University of California, San Francisco Institutional Review Board.

### Primary exposure

The primary exposure was community poverty level. Five-digit zip-codes of the addresses where patients were located at symptom onset were queried. We geocoded these individual zip-codes to link patients to specific census tracts and census blocks. We obtained census tract and block-level socioeconomic variables from the 2015–2019 American Community Survey (ACS) data using the Geographic Information Systems. These geographic units are designed to be homogeneous with respect to population characteristics, economic status, and living conditions [18]. Community poverty level ascertained by utilization of ACS information is based off of percentage of peoples in a given census tract who live below the federal poverty line based on income level. The census-tract level is highly sensitive to gradients in health [8, 19] with poor health outcomes consistently reported in communities where ≥20% of the population live below the poverty level [9–11]. Therefore, community poverty level as a primary exposure was dichotomized as a concentration less than 20% and greater than or equal to 20%.

### Primary outcome

The primary outcome is time from symptom onset to presentation to the pediatric rheumatologist. Date of symptom onset, date of first presentation to a pediatric rheumatologist, and subject’s medical history was obtained from provider questionnaires completed using information from the family and verified by medical records. From these data, the primary outcome of the time from symptom onset to presentation to a pediatric rheumatologist was calculated.

### Study covariates

De-identified demographic information and clinical characteristics from the baseline registry visit were analyzed. Demographic information included patient age, reported race and ethnicity, reported household income, insurance status, highest level of guardian education. Clinical characteristics such as duration of patient-reported morning stiffness, RF antibody status, and disease activity and disability at first pediatric rheumatology visit were queried. Disease activity was calculated using the Clinical Juvenile Arthritis Disease Activity Score (cJADAS) [20]. Disability was assessed with the Child Health Assessment Questionnaire (CHAQ) [21].

Race and ethnicity were self-reported and defined as White, Asian, Black/African American/African or Afro-Caribbean, Hispanic/Latino/Spanish origin, other, and prefer not to answer. Self-reported household income level was defined as <$25,000, $25,000-49,999, $50,000-99,9999, and $100,000+. Parent/guardian education level was defined as high school or less, college (including 1–4-year college, junior college, or technical school), and graduate school. Insurance status was categorized as public insurance (including Medicaid, a state specific children’s insurance plan, and military health care), private insurance, or other (including no insurance, more than one, non-US insurance, self-reported as other, or missing).

### Statistical analysis

Descriptive statistics were generated to describe the study population using N (%) and median (IQR). Kaplan-Meier curves were generated to visualize time from symptom onset to first rheumatologist visit using dichotomized community poverty as the primary exposure. Log-rank tests were used to identify any differences between groups. Cox proportional-hazards models stratified by age at baseline were utilized to estimate hazard ratios for the relationship between community poverty, as a primary predictor, and time to presentation to a pediatric rheumatologist as the defined event of interest or hazard. Analyses were adjusted for demographic features, including sex and race/ethnicity; key variables associated with SES, including insurance and reported family education level; and variables associated with disease characteristics like patient reported morning stiffness and baseline CHAQ to account for disease severity and disability and IgM RF initial to account for more aggressive disease due to seropositivity. Collinearity between patient reported morning stiffness and baseline cJADAS was assessed with a Wilcoxon rank sum test, and morning stiffness was selected in the final model due to missingness of cJADAS values. Statistical significance was evaluated at the 0.05 alpha level, and 95 % confidence intervals were generated for all predictor estimates. All analyses were performed in R Core Team (Survival and Tidyverse, version 4.0.3, 2019, Vienna, Austria).

### Sensitivity analysis

While community poverty level was calculated based on a subject’s zip code of residence at first rheumatologist visit, household income level was self-reported in the Registry. Reported income had a large number of missing data (26.7%). Therefore, we conducted a sensitivity analysis using family reported income as the primary predictor of time to first rheumatologist visit to assess for validation between family-reported income and investigator-calculated community poverty level. This sensitivity analysis was performed for both the Kaplan-Meier analysis and the Cox-proportional hazards model.

## Results

### Baseline characteristics

**Fig. 1:**
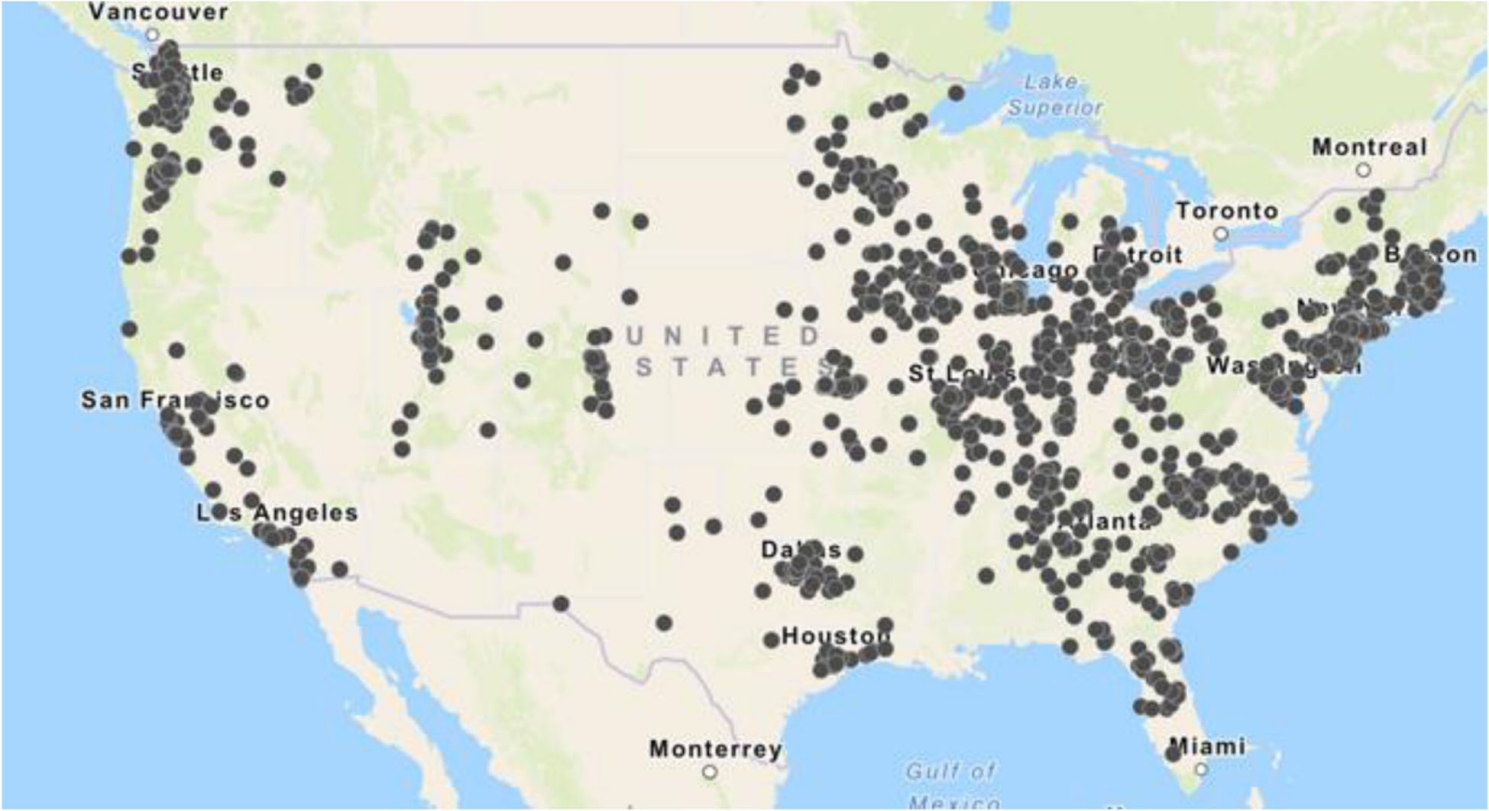
Geographic distribution of the CARRA pJIA patients included in this study

This study sample included 1684 participants who met inclusion and exclusion criteria for our study, out of the 3637 pJIA participants in the CARRA Registry. The majority of those excluded did not meet the requirement of 5 or more active joints within the first 6 months of disease, therefore not meeting ILAR classification criteria for polyarticular JIA or non-residency in the United States. The baseline characteristics of the 1684 included patients are summarized in Table 1. The median age of onset of pJIA was 7 years (IQR 3, 11), 79% were female, 17.6% identified as minority race and/or ethnicity; 19% were from communities with ≥20% community poverty level, and 25.5% utilized public insurance. More than half of the cohort reported a family income greater than $50,000 per year (53.2%) while 27% of the total cohort did not report family income. Guardian education was college level or greater among 56.2% of participants though there was significant missingness also for this key demographic feature (28.4%). The geographical distribution of the study patients in the United States are shown in Fig. 1.

**Table 1:** Demographic and clinical characteristics of pJIA patients enrolled in this CARRA Registry cohort

Characteristic	Total ***N*** = 1684
**Sex**	
Female	1322 (79%)
Male	362 (21%)
**Age at diagnosis – Median (IQR)**	7.0 (3.0, 11.0)
**Race/Ethnicity**	
White	1246 (74%)
Asian	53 (3.1%)
Black, African American, African, or Afro-Caribbean	63 (3.7%)
Hispanic, Latino, or Spanish origin	167 (9.9%)
Other	130 (7.8%)
Prefer not to answer	25 (1.5%)
**Household Income**	
< $25,000	150 (8.9%)
$25,000-49,999	188 (11.2%)
$50,000-99,999	414 (24.5%)
$100,000+	483 (28.7%)
Prefer not to answer or missing	449 (26.7%)
**Community Poverty Level**	
Less than 20%	1368 (81%)
Greater than or equal to 20%	316 (19%)
**Parent/Guardian Education Level**	
High school or less	260 (15.4%)
College (1–4 year college, junior college, or technical school)	656 (39%)
Graduate school	289 (17.2%)
Prefer not to answer or missing	479 (28.4%)
**Insurance**	
Public	430 (25.5%)
Private	1123 (66.7%)
None/Other/Non-US/More than one	131 (7.8%)
**Patient Reported Morning Stiffness**	
< =15 min	1114 (66.2%)
> 15 min	438 (26%)
Missing	132 (7.8%)
**Baseline cJADAS**	7.5 (3.0, 12.0) [2]
Unknown	641
**Baseline CHAQ**	0.25 (0.00, 0.87) [2]
Unknown	130
**IgM RF**	
Positive	309 (18%)
Negative	1204 (71%)
Not performed	171 (11%)
**Anti-CCP**	
Positive	252 (15%)
Negative	786 (47%)
Not performed	646 (38%)

### Clinical outcomes

Median time from onset of symptoms to first pediatric rheumatology visit was 3 months (IQR 1 month, 6 months); 20% of the cohort did not have this information available. Key clinical features obtained included self-reported morning stiffness, of which the majority of the cohort reported none or less than 15 min (66.2%). Median baseline cJADAS was 7.5, though we were unable to calculate for 38% of the cohort due to missing joint counts. Of note, in this cohort, cJADAS and morning stiffness were found to be collinear and strongly associated in distribution with a Wilcoxon rank sum test (*p* < 0.001). Baseline median CHAQ was 0.25.

### Time to first pediatric rheumatologist visit

**Fig. 2:**
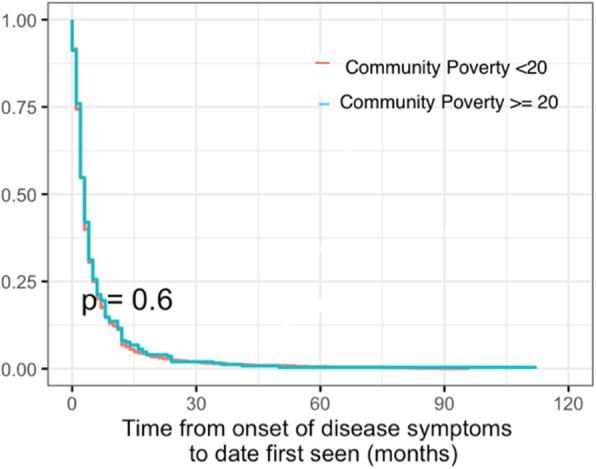
Time from symptom onset to date first presentation to pediatric rheumatologist using community poverty level as the primary predictor

In unadjusted analysis, there was no statistically significant difference (*p* = 0.6) between time to first pediatric visit for newly diagnosed pJIA patients when analyzing by the primary exposure- community poverty level of < 20% versus ≥20%. The median time to presentation to care for both these groups was 3 months (95% CI 2–3 months). Figure 2 demonstrates this Kaplan-Meier time-to-event analysis.

Cox proportional hazards regression yielded an unadjusted hazard ratio of 0.96 (95% CI: 0.84–1.11) when comparing time to first rheumatology visit among patients with ≥20% community poverty level to patients with < 20% community poverty level. Adjusting for sex, race/ethnicity, insurance, reported family education level, patient reported morning stiffness, RF status, and baseline CHAQ score, yielded a hazard ratio of 0.81 (95% CI: 0.67–0.99, *p* = 0.038) when comparing time to first rheumatology visit among patients with ≥20% community poverty level to patients with < 20% community poverty level. This suggests that patients living in areas with a ≥ 20% community poverty level were 19% less likely to be seen by a rheumatologist compared to those living in < 20% community poverty level at the same time point. Therefore, those individuals living in higher poverty areas experienced delays in first presentation to a pediatric rheumatologist.

In the multivariate Cox analysis (Table 2), public insurance was not a significant predictor of delay in seeing a rheumatologist (HR 1.01, 95% CI 0.84–1.21, *p*-value > 0.9). Additionally, guardian education level was not found to be a significant predictor (high school or less: HR 0.96, 95% CI 0.75–1.23, *p*-value = 0.7; college or junior school: HR 0.97, 95% CI 0.81–1.17, *p*-value = 0.8; graduate school as referent).

**Table 2:** Cox proportional hazard estimate of effect of community poverty level, demographics, and disease characteristics on time to first rheumatologist visit

		Adjusted^**1**^	
Characteristic	HR^**2**^	95% CI^**3**^	***p***-value
**Community Poverty Level**			
< 20%	–	–	–
≥ 20%	**0.81**	**0.67–0.99**	**0.038**
**Sex**			
Male	–	–	–
Female	1.06	0.88–1.27	0.600
**Race/Ethnicity**			
White	–	–	–
Asian	1.14	0.73–1.77	0.600
Black, African American, African or Afro-Caribbean	1.41	0.91–2.16	0.120
Hispanic, Latino, or Spanish origin	**1.33**	**1.01–1.76**	**0.043**
Other	1.21	1.21–4.10	0.200
Prefer not to answer	**2.23**	**1.21–4.10**	**0.010**
**Insurance**			
Private	–	–	–
Public	1.01	0.84–1.21	> 0.90
**Reported Family Education Level**			
Graduate School	–	–	–
College, Junior College, or Technical School	0.97	0.81–1.17	0.800
High School or Less	0.96	0.75–1.23	0.700
**Patient Reported Morning Stiffness**			
≤ 15 min	–	–	–
> 15 min	1.12	0.93–1.36	0.200
**Anti-CCP**			
Negative	–	–	–
Positive	0.99	0.73–1.35	> 0.90
Not Done	1.04	0.88–1.24	0.600
**IgM RF**			
Negative	–	–	–
Positive	0.94	0.70–1.25	0.700
Not Done	1.14	0.79–1.65	0.500
**Baseline CHAQ**	0.91	0.80–1.03	0.140

1: Cox-proportional hazard model stratified by age at baseline (> 9 and < =9) adjusting for sex, race/ethnicity, insurance, reported family education level, patient reported morning stiffness, anti-CCP, RF IgM, and baseline CHAQ

2: Hazard ratio

3: Confidence interval

4: Child Health Assessment Questionnaire

Unadjusted sensitivity analysis using family reported income level as the primary predictor demonstrated no statistically significant differences between income groups with time to first rheumatology visit (*p* = 0.13) using Kaplan- Meier analysis. Adjusted sensitivity analyses did not demonstrate a statistically significant difference in time from symptom onset to first visit across all family reported income levels (($50–99,999: aHR 0.97, 0.79–1.18, *p*-value 0.7; $25–49,999: aHR 0.91, 0.66–1.26, *p*-value 0.6; <$25,000: aHR 0.99, 0.69–1.43, *p*-value > 0.9; reported income of ≥$100,000 as referent).

## Discussion

In adjusted analysis for this study, children with new-onset polyarticular JIA residing in neighborhoods with ≥20% community poverty experienced delays in presentation to a pediatric rheumatologist as compared to children with pJIA residing in neighborhoods with lower levels of community poverty. Importantly, the impact of community poverty on delay to rheumatology care was seen even while adjusting for other known social determinants of health, including insurance status and guardian education level (used as a surrogate for health literacy). Such covariates may have acted as negative confounders in the unadjusted model, accounting for the absence of association that was subsequently identified upon adjustment in the multivariate Cox regression analysis. The presence of delays despite adjustment for other social determinants suggests the direct effect of community poverty on delays to care not mediated through other SDoH.

Several intermediate events must occur during the time from symptom onset to diagnosis, and ultimately treatment [14–16]. These events include the recognition of symptoms, visit to a primary care provider, time to referral to a specialist, and time between referral and attending a specialist appointment. While the data used in this study could not truly reflect the steps in this process to help decipher where there was greatest delay, they suggest that community poverty may lead to delays in the overall process of presenting to subspecialty care. Potential factors contributing to delays could include absence of a regular medical home/primary care, lack of transportation, inability to afford costly co-pays associated with subspecialty care, and inability for families to miss work for scheduled appointments. Steps in this process amenable to intervention are critical to identify.

It is worth noting that the pJIA CARRA Registry cohort is unlikely to reflect the full diversity of the population of children with pJIA in the US. Our pJIA cohort was comprised of 74% White children (compared to 81% in 2017 and 93% in 2013) [22, 23] with 20% reporting an income level < $50,000/year (compared to 8.9% in 2013) [22]. A recent publication from the Children’s Hospital of Philadelphia has shown more racial diversity in their pJIA cohort, including a higher proportion (13%) [24] of Black patients (compared to 3.7% in our cohort). Overall, the demographic characteristics of our cohort of the CARRA Registry patients are predominantly White, affluent, educated, and privately insured. Our participants’ families were more educated than average (56.2% with college degree or higher versus 32% in the average US population [25]), had higher wealth (53.2% of the cohort reporting at least $50,000/year income compared to the US median income of $62,843 [25]), and had lower rates of public insurance (25.5% versus 35% among all US children [26]). A publication from Cincinnati Children’s showed that Medicaid insurance was utilized most often by non-White children with JIA (*p* = 0.04) [27]. The paucity of minority recruits in the CARRA pJIA cohort might explain discrepancies in the percentage of children with public insurance in our study. In the 2015–2019 ACS 5-year estimates, 21.1% of the population lived in census tracts with poverty rates of 20% or higher with a sizable portion of these communities in the Midwest and the South [28]. In our cohort, 19% of patients lived in communities with ≥20% community poverty and as seen in Fig. 1, most patients resided on the East and West coasts of the US.

The absence of diversity in our cohort highlights the need to prioritize and improve diverse patient enrollment in pediatric registries to improve generalizability of research results and to be able to more accurately understand the relationship between social determinants of health, including socioeconomic status and racial disparities, and disease outcomes. From a public health perspective, it is imperative to ensure early referral and intervention for children across different socioeconomic statuses to promote equal and timely access to quality health care.

### Strengths

The strength of this study is the large sample of patients from the CARRA Registry. An additional advantage was our retrospective cohort study design which allowed for time-to-event analyses and calculation of hazard ratios as many prior studies of socioeconomic status in JIA have been cross-sectional in nature with interpretation limited to association.

### Limitations

While this CARRA cohort may not be demographically representative of the overall pJIA population as described previously, the large sample size has given us the opportunity to study the effect of community poverty as a potentially modifiable key step in the long-term care of these patients. Furthermore, we expect that the lack of diversity would bias our study towards the null, and therefore does not threaten the validity of our findings.

We encountered significant missingness in our dataset for key variables such as household education level (28.4%) and predictors such as reported income level (26.7%). Our sensitivity analysis using reported income demonstrated no major differences in time to first pediatric rheumatologist visit on Kaplan-Meier analysis and the Cox model. Importantly, due to the reluctance of lower income earners to report financial details, this was likely a nonrandom nonresponse [29]. Therefore, this may not reflect the true underlying relationship between income level and time to first rheumatology visit. To control for baseline disease activity, morning stiffness of less than or equal to 15 min was used due to inability to calculate the cJADAS for a significant portion of our cohort. Morning stiffness of less than or equal to 15 min may not reliably discern mild, moderate, or severe disease activity at disease onset; therefore, there is the possibility of residual confounding in our model such that cautious interpretation is suggested.

Finally, this analysis does not address the role that SDoH may play in longitudinal disease activity or disease damage which will be the subject of future work.

## Conclusion

Our study found evidence of community poverty as a predictor of delayed time to rheumatology care in newly diagnosed pJIA patients. Further studies should focus on the intersection of economic deprivation and racial disparities, which have been previously suggested in studies from an earlier iteration of the CARRA Registry [22, 23]. Further identification of specific barriers that delay presentation to a pediatric rheumatologist may inform critical public health policy change to minimize delays to care and thus improve pJIA disease outcomes.

## Data Availability

The data that support the findings of this study are available from the CARRA Registry but restrictions apply to the availability of these data, which were used under license for the current study, and so are not publicly available. Data are however available from the authors upon reasonable request and with permission of the CARRA Registry.
